# Philadelphia County Medical Society

**Published:** 1890-09

**Authors:** 


### PHILADELPHIA COUNTY MEDICAL SOCIETY.

*Regular Meeting, June 25, 1890.*

The president, Dr. John B. Roberts, in the chair.

### THE RECOGNITION OF EYE-STRAIN BY THE GENERAL PRACTITIONER.

By EDWARD JACKSON, M. D.,

Professor of Diseases of the Eye in the Philadelphia Polyclinic.

The attempt to give relief from the symptoms of eye-strain by a careful trial, seriatim, of one's favorite sedative, tonic, and alterative prescriptions, followed by experimentation with the formulæ of great professors found floating on the surface of medical journalism, does not usually bring much comfort to the patient or credit to the doctor. And that it is so frequently persisted in until the patient deserts his so-called medical adviser, and of his own notion takes his chances with the specialist or the charlatan, seems to argue an inability to recognize the connection of this group of symptoms with their cause. The worst evil of specialism is ignorance and indifference as to other departments of medicine; one of the most aggravated manifestations of this evil is the expressed indifference of so-called "general practitioners" toward the anomalies and diseases of the eye.

From time to time efforts have been made by ophthalmologists to secure a more general recognition of eye-strain on the part of the mass of the profession; but usually these efforts have consisted in a recommendation of some special instrument or procedure of diagnosis, as the refraction ophthalmoscope, or the shadow-test, or a set of trial lenses reduced in size and price to the supposed needs of the mass of the profession. If it were really necessary to apply such special means of diagnosis in order to recognize the presence of eye-strain, there would be little prospect of its early general recognition. But it is frequently recognized by the patient himself, and the ophthalmic surgeon finds in the general rational symptoms quite sufficient grounds for a provisional diagnosis; hence, if the mind is clear from preconceived hypotheses as to the causes of the symptoms, tending to divert attention from their real origin, there is no reason why anyone respectably qualified for general practice of medicine should not be able to make a provisional diagnosis with sufficient certainty to serve for the basis of further investigation and treatment, in the great majority of cases, without resort to any special method of examination whatever. Of course, the ophthalmoscopic evidence of ametropia, when it can be obtained, is very valuable as confirming such a diagnosis; and I do not underestimate the value of the ophthalmoscope to the general practitioner, for I cannot regard anyone who is unable to use the opthalmoscope as properly qualified for general practice. But I do say that inability to measure refraction with the ophthalmoscope is no reason for failing to recognize eye-strain.

The patient suffering from eye-strain comes with a certain history and certain complaints, which, carefully considered by the light of a very moderate knowledge of the subject, clearly indicate the cause of the trouble, in the great majority of cases. The symptoms in question may be considered separately.

1. *Impairment of vision*, either quite temporary, more prolonged, or quite permanent. A very characteristic form of temporary impairment of vision is that due to sudden relaxation of the accommodation. This occurs when the ciliary muscle has long been overtaxed, and especially in the latter hours of the day, when it is nearly tired out. The patient notices that the print or other near object on which the attention is fixed suddenly becomes entirely blurred, compelling the cessation of the eye-work. After a moment, however, the power of again focusing the object returns, and work can be resumed. The patient is apt to close his eyes for an instant, and, perhaps, rub them, and on again opening them finds the sight again restored. If the eye-work is continued, the failure of accommodation recurs, to again rapidly pass away; and keeping on with the eye-work, these periods of inability to see become more and more frequent, until, finally, they greatly interfere with the continuance of the work or quite prevent it. This form of impairment affects only the vision for near work.

Another temporary impairment is that due to spasm of the accommodation; it affects distant vision only, and is noticed chiefly by those whose distant vision is otherwise pretty good. It comes on after prolonged straining of the eye, usually for near vision, and lasts until the eye has become well rested. It is a valuable danger signal, and should secure cessation from the work which produced it until it has given place to normal relaxation. Permanent impairment of vision is brought about when eye-strain causes myopia or decided permanent damage of the choroid and retina.

2. *Headache and aching of the eyes.*—Eye-strain should be the first thought suggested by any complaint of headache, for in our day and civilization it is by far the most common cause of that symptom. It enters as a factor into the causation of nearly all headaches not due to pyrexia, toxemia, or diseases of the brain or its membranes. The simple existence of headache, therefore, should suggest eye-strain; but frequently a careful inquiry as to the manner and time of occurrence of the attack, and the location of the severest pain, will be almost conclusive as to the origin of the trouble.

Often it comes on whenever the eyes are used, and is absent when they have had a proper period of rest. The occasions of most severe requirement in the direction of eye-work are the doing of anything requiring accurate near vision, taxing both the accommodation and the convergence; or traveling, shopping, attendance at public gatherings, which entail more use of the eyes than the patient is at the time conscious of, and often under unfavorable conditions.

Very often the chronological connection between the use of the eye and the occurrence of the ache, although perfectly certain and evident when once it has been observed, has never been noted by the patient until his attention has been directly called to it. Even when the headache seems constant and quite uninfluenced by variations in the amount of eye-work, it may be due wholly to eye-strain.

In hyperopia in young people the accommodation is in excessive use so long as the eyes are open and the attention fixed on any visible object; and hyperopia is the most common cause of constant headache. The writer was formerly subject to a constant headache whenever confined to the house, and regarded it as caused by breathing vitiated air, until it was quite cured by the correction of his hyperopic astigmatism. Many persons have the same idea as to the causation of the headaches they always experience when attending the theatre or other place of public amusement, and which are really due to eye-strain. Others ascribe these headaches to exhaustion, especially those experienced in traveling or shopping. This is nearer the truth, only they commonly have in mind a condition of general exhaustion, whereas it is largely one of local exhaustion of the special nervous apparatus concerned in the act of seeing.

The *location of the aching* is of some significance. Generally it is frontal, often described as beginning in the eye, or just back of the eye, or through the temples. Frequently it extends to the occipital region, and may sometimes be felt principally or wholly in that region. Headache most severe in the vertex or confined to that region is probably not very common from any cause, but from eye-strain it is almost unknown. Often the headache is more severe on one side of the head than the other. Sometimes it is entirely confined to one side, but usually it is bilateral.

Those more or less regularly periodical headaches, known as nervous or sick headache, migraine, or, when confined to one side of the head, hemicrania, are in many cases set up by eye-strain and relieved by its removal. Attacks of this kind are frequently ushered in by certain interference with vision and subjective sensations of light, affecting a part or the whole of the visual field, and known as ophthalmic migraine. These visual disturbances are simply a part of the general "nerve-storm," and it is not certain that they especially indicate the origin of the attacks to have been eye-strain.

3. *Congestion, irritability, or inflammation of the eyes and their appendages* should always suggest the suspicion of eye-strain.

A single attack or manifestation of this kind has no especial significance, but repeated attacks of inflammation, or prolonged congestion or irritability, are exceedingly suggestive of a continuing cause ; and the most common of these is the one now under discussion. No case of chronic inflammation of the margins of the lids, or of recurring conjunctivitis, or repeated styes, has justice done to it until it has been carefully investigated for eye-strain. Persons at the period when they begin to feel the effects of loss of accommodation in presbyopia or absolute hyperopia, suffer from repeated attacks of conjunctivitis, which they commonly ascribe to "taking cold in the eye," but which are cut short by use of the appropriate lenses, and which, if unchecked, would tend to establish a chronic catarrhal condition, which is a chief discomfort in the lives of many elderly people.

Of course, these conditions of ocular congestion and inflammation will be recognized by the usual symptoms of redness, swelling and itching, smarting or burning pain. They often require especial local treatment, and will quite often be temporarily cured by this alone ; but if the underlying cause is not removed, they show a strong tendency to recur indefinitely, or until the accommodation is so far lost that the temptation to strain it is removed. It should be noted that usually headache and these local inflammatory conditions are not presented by the same case. They may co-exist, but, more commonly, if one is decidedly present, the other is absent.

So far, nothing has been mentioned for the diagnosis of eye-strain but the facts ascertained by questioning the patient and from simple inspection of the eye. If, now, the physician's office contains — what every general practitioner's office should contain — a card of test letters for accurately ascertaining the distant vision, and a card of fine print for ascertaining the near point of the eye, additional valuable evidence is easily obtainable. The trial of the distant vision will give indication of any considerable degree of myopia or astigmatism. But it must always be borne in mind that troublesome ametropia may be present without preventing perfect distant vision. The position of the near point, if farther from the patient's eye than his age would indicate, is pretty good evidence of the strain of the accommodation. Evidence of strain of the external muscles of the eye, heterophoria, can be obtained by simply getting the patient to keep his eyes fixed on some object, near or distant, and covering one eye, then noting whether the covered eye deviates from its position of fixation, and especially whether it makes a quick movement to return to that position when it is uncovered.

To recapitulate, briefly, the common symptoms of eye-strain are :

1. Certain forms of impairment of vision.

2. Headache, which is to be studied with reference to the times of its occurrence and the parts of the head to which the aching is referred, with careful discrimination between the patient's facts and his theoretical explanation of them.

3. Chronic or repeatedly recurring congestion, or inflammation of the eye or its appendages.

And, if, to these symptoms are added the results of the simple tests of near and distant vision, and evidence of tendency of the eyes to deviate from their normal position when covered, a very good basis is furnished for the probable or provisional diagnosis of eye-strain, without recourse to any special apparatus or unusual diagnostic procedure. And, in view of these facts, there is no justification for the general practitioner who fails to recognize most of the numerous cases of eye-strain with which he is brought in contact.

DISCUSSION.

Dr. George M. Gould : Certainly nothing Dr. Jackson has advanced in his excellent paper calls forth controversy or criticism ; but I think a word may be added as to certain other symptoms not alluded to by him, that may sometimes put the general practitioner on the track of an eye-strain reflex. When eye-strain is sufficiently severe to set up a reflex neurosis, sleepiness is a common symptom brought on by persistent reading or writing. The patient cannot understand why he grows so drowsy. A more important trouble is one I at first advanced somewhat doubtfully, but now, I am growing perfectly convinced, is a genuine result of ametropia. I allude to troubles of appetite and digestion. Few patients with severe or long-continued eye-strain that do not complain of anorexia, fickle appetite, or some dyspeptic trouble. Mothers frequently call such girls "pickers." Explain the mechanism of this reflex as we may, I am sure it is a fact, and that a malnutrition often results that may end in anemia and many different forms of nervous abnormality. I have had a large number of such patients regain long-lost appetite after putting on glasses, and regain ten to twenty pounds of flesh within a month or two. Nervousness and choreic movements, even genuine choreas, are traceable sometimes to the same causes. I had one patient who wore the right shoe out in a few weeks, and who had a habit of bursting out crying or into a rage at a trifle. She had been treated for chorea for years at one of our best hospitals. All symptoms have disappeared for two years upon correction of her hyperopic astigmatism. I could cite several other cases.

I should like also to call attention to car-sickness in connection with eye-strain. I have had eight or nine cases of this kind, and by glasses all have been relieved of the car-sickness. One case was that of a gentleman who had car-sickness every journey. While he had the mydriatic in his eyes he went to Washington, and suffered no inconvenience whatever. Subsequently, after he had glasses, he made a trip to St. Paul without any of the former trouble. In the last two days I have had two cases — one that of a girl who could not ride a short distance in the street cars without vomiting. I found a decided degree of hypermetropic astigmatism. With the mydriatic in her eyes she rode home without her usual trouble.

A strange thing with reference to eye-strain is, that it often exists to an exceptional degree without showing any symptoms in the eye. The patient will often say that the eyes are perfectly good and have never caused any irritation. The reflexes seem to settle in some other place. This is an interesting pathological and physiological question.

With reference to testing the eyes by the general practitioner, it has struck me that a simple plan, which could be readily carried out, would be as follows: Have two test-cards, so that the patient will not learn and remember the letters. Let him first test distant vision with one of the test-cards; then let him instil homatropine. This will give perfect paralysis in three-quarters of an hour. Now retest with the other card. Then, if vision has decreased, there is eye-strain due to astigmatism or hypermetropia. Another practical point is that, if the patient is suffering with headache, he will be relieved by the application of the mydriatic. It is as to the existence of hyperopia and astigmatism that we want to know. Myopia rarely produces eye-strain.

Dr. Mary E. Allen: I would ask if, in these cases, the condition of the recti muscles has not something to do with the symptoms. In my own case I suffered eye-strain for a long time with insufficiency of the recti muscles. I had one symptom which I have never seen described, and that is a feeling as though a blow had been struck against the eye. My explanation of this is that, by a spasmodic contraction of the straight muscles, the elastic eye-ball is suddenly drawn with force against the back of the orbit, giving the sensation of a blow. I had this a long time before wearing glasses, but very seldom since.

Dr. Jackson: My paper simply refers to the *recognition* of eye-strain. I purposely considered only those symptoms most generally present, and had in mind the great mass of cases, not the exceptional ones, which do, in the aggregate, constitute a very large number, but still are proportionately few. The other symptoms which have been mentioned, and many others, might be referred to as due to eye-strain, but they do not occur in the large number of cases, and can hardly be regarded as of general importance in making the probable diagnosis.

I have used the term eye-strain, not as a synonym of ametropia, because we may have ametropia without eye-strain, or eye-strain without ametropia, if requirements are put upon the eye too great for its capacity. You can have it without any ametropia or weakness of any extra-ocular muscle or group of muscles. It is to the question of eye-strain as isolated and separated from ametropia that I refer. Of course, in a very large proportion of cases, the relief of the eye-strain comes from correction of the ametropia.

In connection with that correction, I should like to say one thing with reference to a remark made by Dr. Gould — that is, if myopia were present it need not be considered. In my experience, myopia may cause severe eye-strain. There is the strain of convergence, and any inequality between the two eyes in the amount of myopia — and myopia is usually unequal — is very likely to cause eye-strain. The discovery of myopia would not, to my mind, rule out the existence of eye-strain.

